# Supplementary figures and images for: An Integrated Bioinformatics Study of a Novel Niclosamide Derivative, NSC765689, a Potential GSK3β/β-Catenin/STAT3/CD44 Suppressor with Anti-Glioblastoma Properties
Source: Int J Mol Sci. 2021 Feb 28;22(5):2464. doi: 10.3390/ijms22052464 (PMC7957701; doi:10.3390/ijms22052464)

## Slide 1
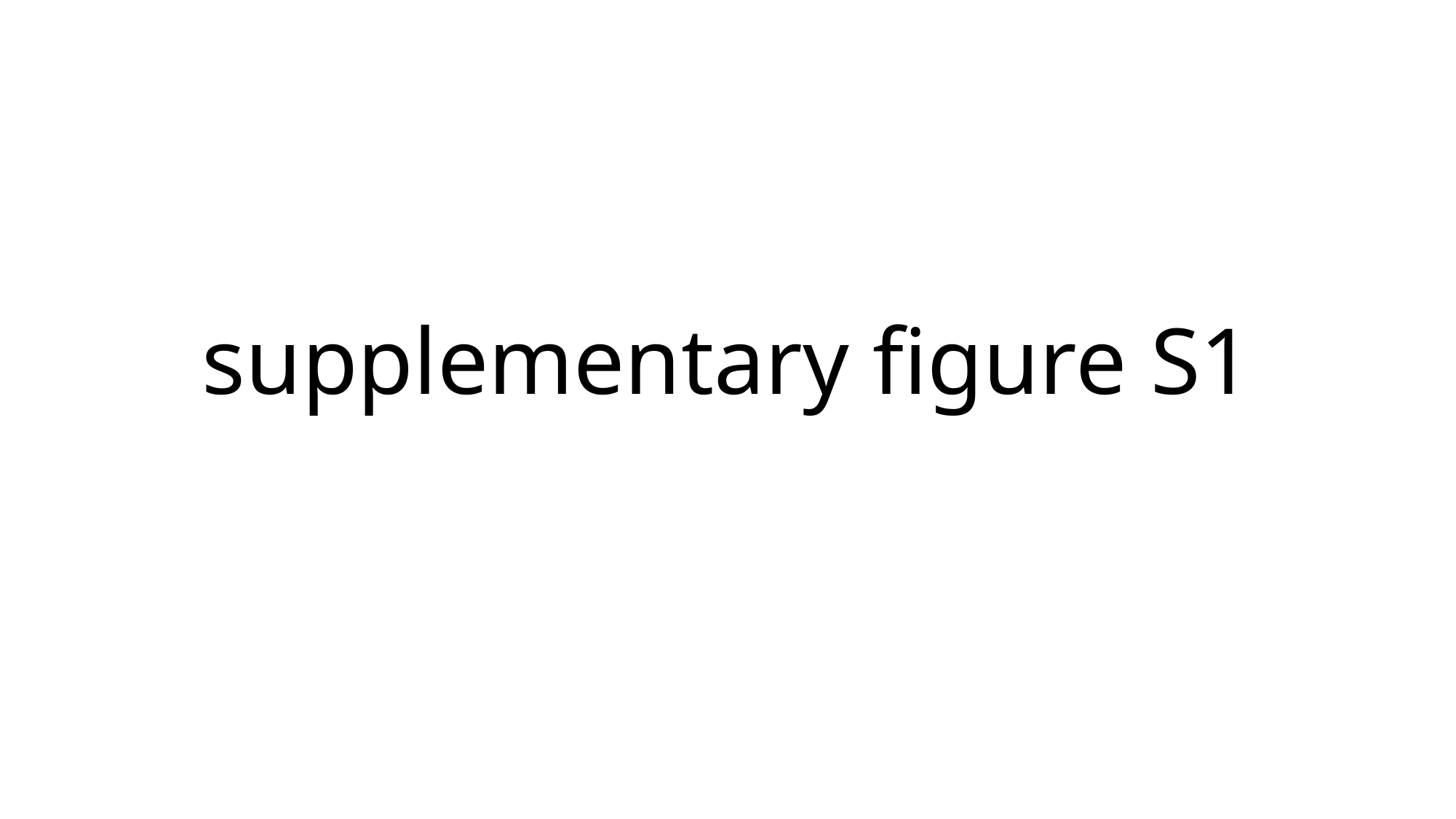

# supplementary figure S1

## Slide 2
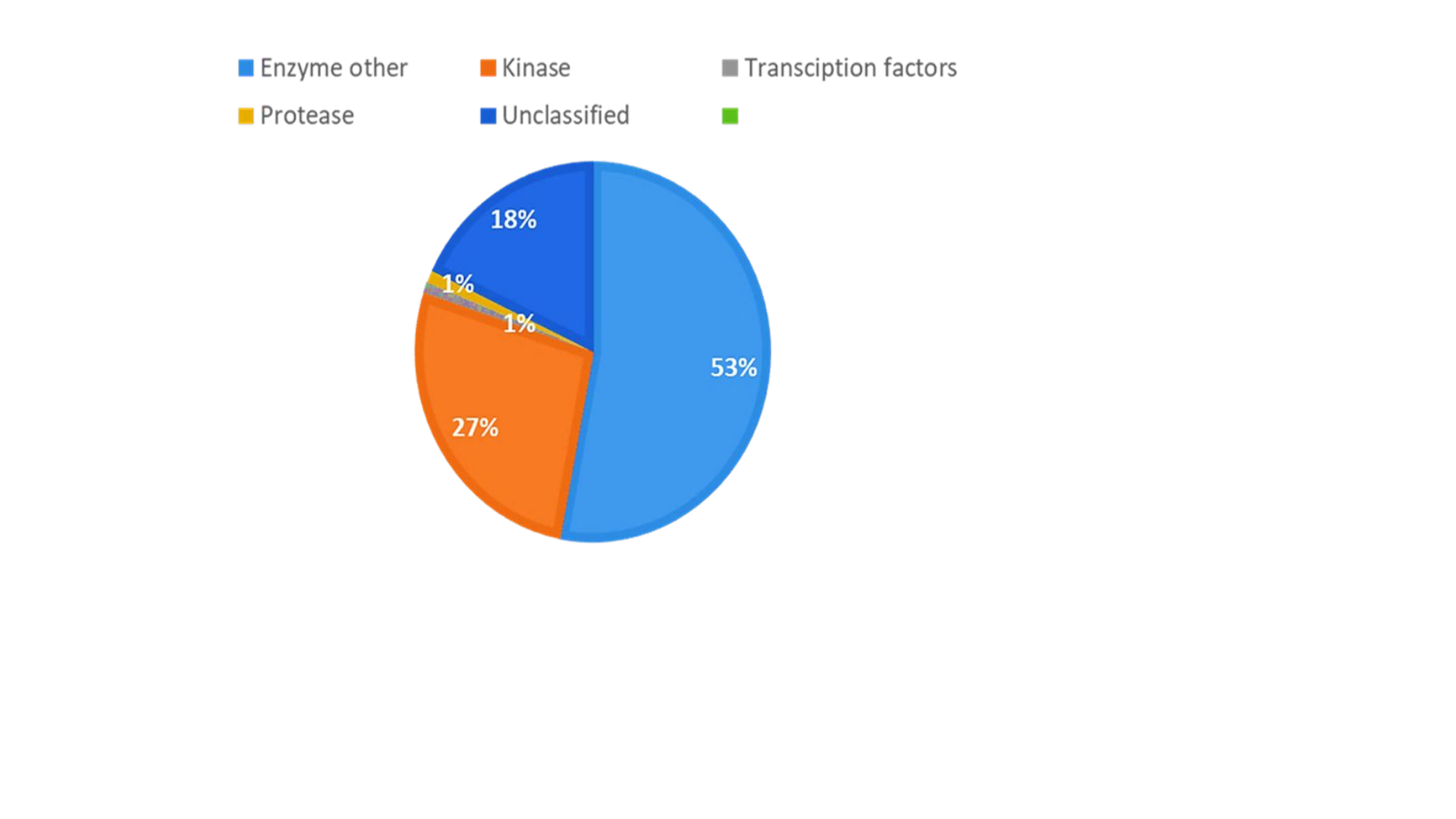

Supplement: Supplementary file 1 [file ijms-22-02464-s001.zip › ijms-1106197-supplementary.pptx]
